# First insights of integrating the Bonn Internship Curriculum for Point-of-Care Ultrasound (BI-POCUS): progress and educational aspects

**DOI:** 10.1186/s12909-024-05904-2

**Published:** 2024-08-19

**Authors:** Elena Höhne, Valentin Sebastian Schäfer, Simon Michael Petzinna, Agnes Wittek, Jennifer Gotta, Philipp Reschke, Florian Recker

**Affiliations:** 1 Department of Diagnostic and Interventional Radiology, Clinic for Radiology and Nuclear Medicine, University Hospital Frankfurt, Frankfurt, Germany; 2 Department of Obstetrics and Prenatal Medicine, University Hospital Bonn, Bonn, Germany; 3 Department of Rheumatology and Clinical Immunology, Clinic of Internal Medicine III, University Hospital Bonn, Venusberg Campus 1, 53127 Bonn, Germany

**Keywords:** Ultrasound, Training, Education, Undergraduate Education, Curriculum Development

## Abstract

**Background:**

Point-of-care ultrasound (POCUS) is rapidly gaining prominence in various clinical settings. As its use becomes more widespread, there is a growing need for comprehensive ultrasound training in medical education to ensure that future healthcare professionals are proficient in this essential diagnostic tool.

**Objective:**

This study is the first attempt by the University of Bonn to seamlessly integrate ultrasound courses and the use of ultrasound devices into the regular activities of final year medical students and to evaluate the usage of these devices.

**Methods:**

A total of forty students in their practical year were provided with a hendheld ultrasound device for a period of four months. During this time, they were invited to take part in eight optional ultrasound courses in which they acquired images and those images were rated using a specially developed rating system. At the end of the tertial, students were able to take part in a voluntary survey on the use of the equipment.

**Results:**

Participation in the optional ultrasound courses was well received, with the Introduction and FAST module drawing the largest number of participants (29). Among the ultrasound images acquired by students, those of the lungs obtaining the highest rating, with 18.82 (SD ± 4.30) points out of 23 points, while the aorta and vena cava images scored lowest, with an average of 16.62 (SD ± 1.55) points. The overall mean score for all images was 17.47 (SD ± 2.74). Only 21 students responded to the survey. Of the participating students, 67% used the device independently four times or fewer during the tertial.

**Conclusion:**

The study aimed to enhance the BI-POCUS curriculum by improving students' ultrasound skills during their practical year. However, device usage was lower than expected, with most students using it only once a month or less. This raises concerns about the justification of the effort and resources. Future initiatives will focus on technical improvements, better login data provision, and closer monitoring of usage and progress, emphasizing the need for practical ultrasound training in medical education.

**Supplementary Information:**

The online version contains supplementary material available at 10.1186/s12909-024-05904-2.

## Background

Recently, the role of ultrasound in medicine has increased rapidly, as it is mobile, fast and easily accessible. Consequently, ultrasound is fast becoming a pivotal diagnostic instrument across medical specialties, taking the lead as the most widely used imaging tool in clinical practice [1]. Especially point-of-care ultrasound (POCUS) has gained popularity in various clinical environments due to numerous studies proving its benefits in patient care [2–5].The demand for a comprehensive integration of ultrasound into medical training is gaining momentum [6, 7]. However, the introduction of ultrasound into medical education poses challenges. The lack of international consensus and guidelines [8, 9] on its incorporation into traditional curricula and skills testing leaves faculties to determine the extent of teaching. Despite the emergence of the EFSUMB statement recommending the integration of students' ultrasound training into both the preclinical and clinical curricula [10], surveys still reveal a significant gap in the incorporation of ultrasound teaching in the pre-clinical curriculum of European universities [11]. To date, ultrasound courses have not been systematically integrated longitudinally into the basic medical training of all students at our university. In the students ‘ perspective, the primary obstacle lies in the inadequate time allocated for ultrasound in the proposed curriculum and the absence of courses offered by the medical faculty [12]. To close this gap, we have developed a comprehensive curriculum that provides all medical students in their final year with practical knowledge and basic skills focusing on point-of-care applications. Klicken oder tippen Sie hier, um Text einzugeben.In this study, we examine the initial implementation of the Bonn Internship Curriculum for Point-of-Care Ultrasound (BI-POCUS) [13]. This initiative is a step towards addressing the underappreciated value of ultrasound education, emphasizing its benefits as a radiation-free, widely available diagnostic tool relevant to various medical specialties.

## Methods

In Germany, medical students are required to undertake a clinical internship, known as the "practical year," during their final year of medical education. This internship is segmented into three rotational periods, commonly referred to as tertials. Typically, two of these tertials focus on internal medicine and surgery, respectively, while the third tertial allows students to elect a specialization of their choice.

**Fig. 1:**
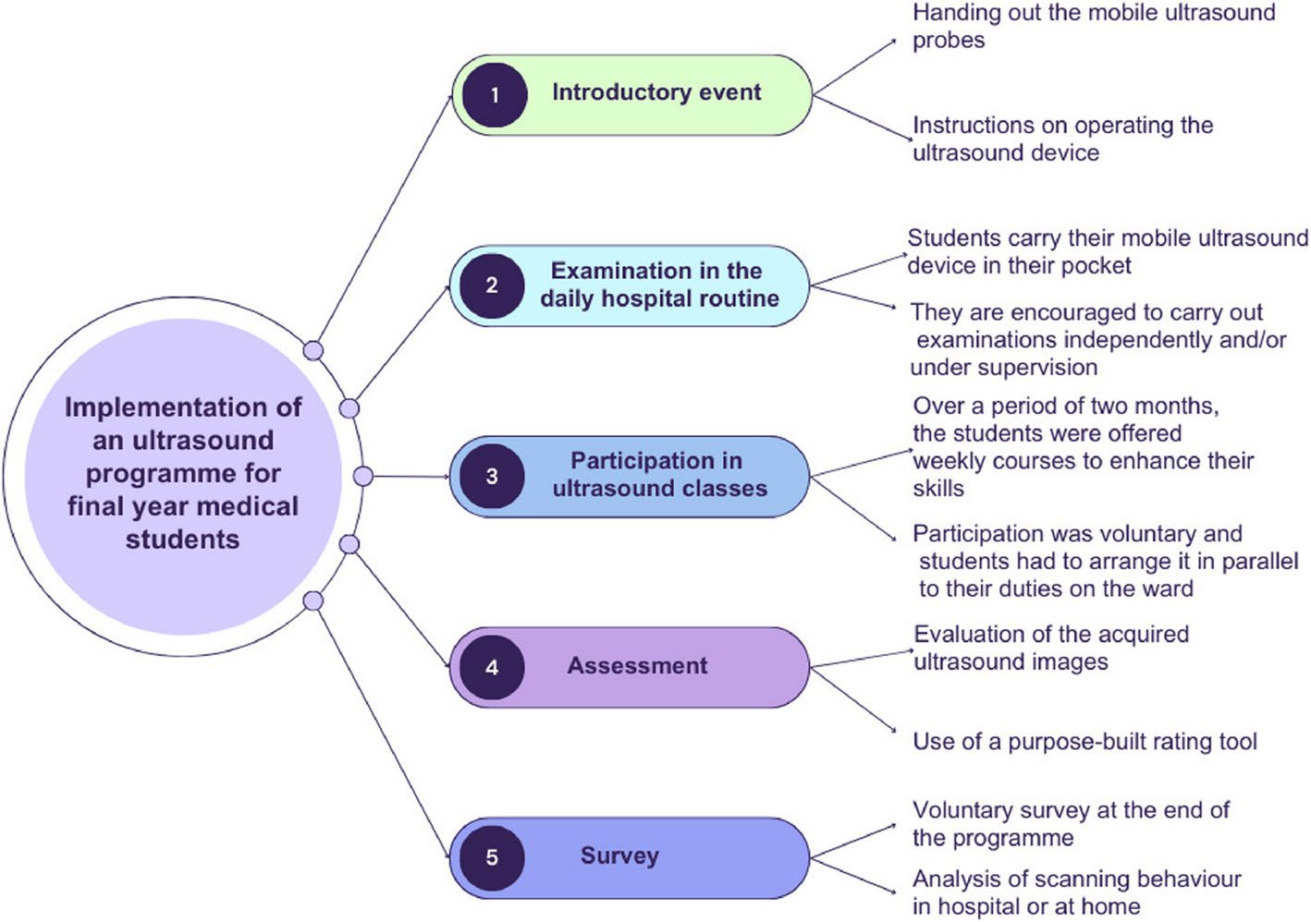
Overview of the ultrasound course program for medical students in their practical year (final year)

At the commencement of the practical year in late 2023, each student beginning a tertial at the University of Bonn was issued a ButterflyIQ ultrasound probe [14] with unrestricted access for the duration of the tertial (Fig. 1). Concurrently, these students were provided with an iPad and the essential credentials required to access the associated software application. Within each clinical department, designated personnel were responsible for enabling the activation of these login credentials.

During an introductory session, the technical aspects of the ultrasound device were explained, and the use of the accompanying app was demonstrated.

**Fig. 2:**
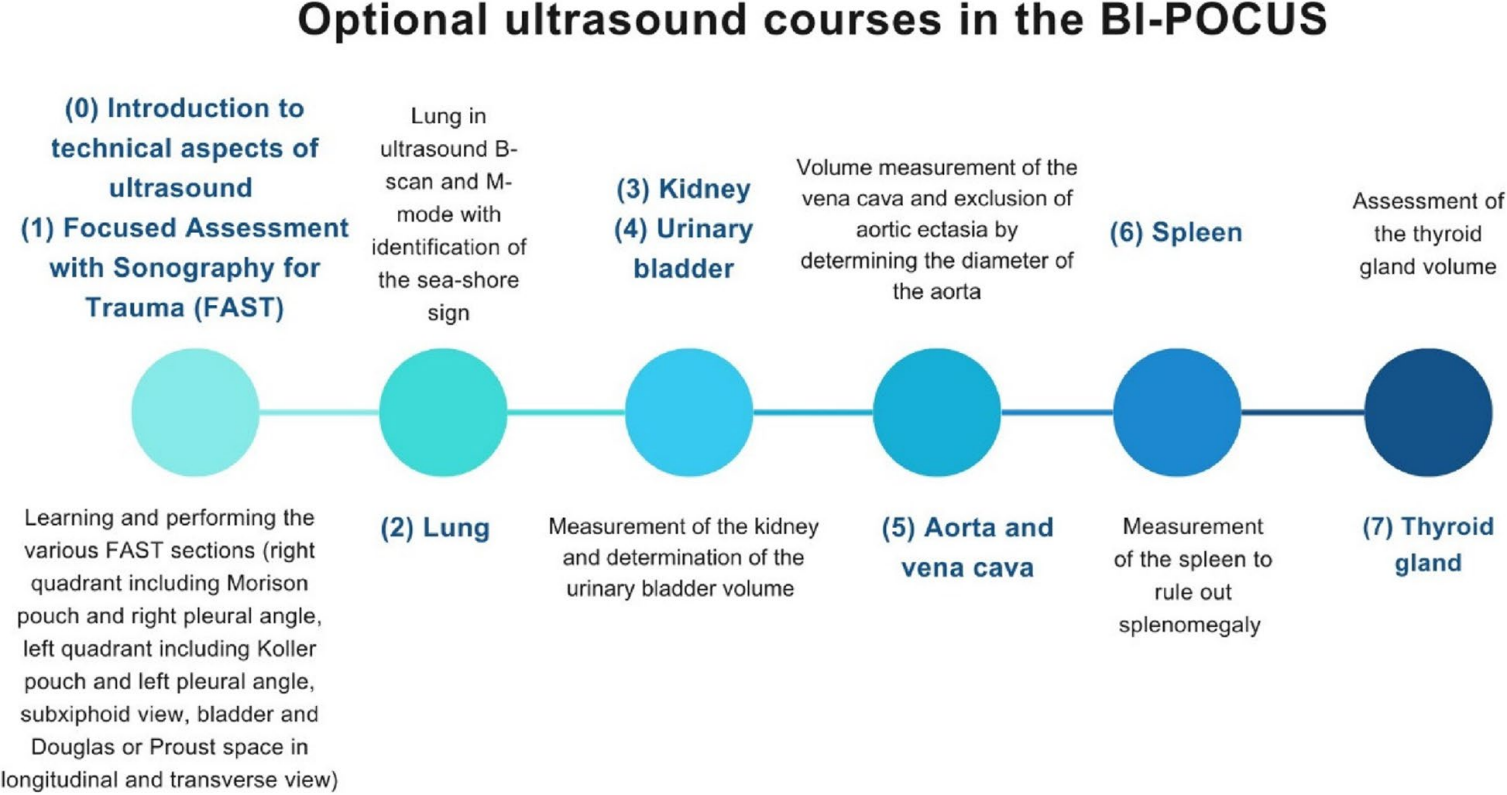
Illustration of the timeline and various ultrasound courses offered to students as a voluntary program during their practical year

In addition to the opportunity of carrying out examinations independently, optional ultrasound courses were offered over a period of eight weeks. The courses were organised by two German Society for Ultrasound in Medicine (DEGUM) certified physicians (level I and level III). The selected modules are part of the BI-POCUS curriculum (general training I and II) developed specifically for medical students in the year of the internship [13]. This curriculum aims to ensure that each student acquires basic skills by the end of the clinical placement year, focusing on globally recognised standard protocols [15]. The eight offered courses are displayed in Fig. 2.

The lessons were offered on an optional basis and conducted during daytime hours, necessitating students to allocate time for participation alongside their responsibilities on the ward. As a result, the composition of participants varied across each course, reflecting the diverse schedules and commitments of the students involved.

A deliberate decision was made not to conduct competence assessments through image rating for the introductory module and the Focused Assessment with Sonography for Trauma (FAST) module. This decision was primarily influenced by the understanding that students required sufficient time to acquaint themselves with the ultrasound device and its technical functionalities before undergoing any formal assessment. The introductory period was deemed essential for building foundational skills and confidence in handling the equipment.

Our educational approach placed a strong emphasis on conducting organ-specific assessments. However, implementing this focus within the FAST module proved to be challenging. The complexity of achieving standardized, high-quality assessments in the context of a fast-paced, trauma-focused module presented significant obstacles. Additionally, the lack of a universally adopted standardized assessment format for ultrasound images in the educational setting further complicated this task.

Recognizing this gap in standardized assessment, we deemed it essential to devise our own evaluation format. Unlike some existing formats that delve extensively into technical intricacies, our approach aimed to simplify the assessment process by leveraging the capabilities of the Butterfly app. This application allows users to effortlessly select the appropriate preset, with key parameters such as frequency and depth being automatically configured. Despite this automation, manual adjustments for depth and gain are still available, providing users with flexibility and control.

In alignment with these functionalities, we meticulously curated the categories integrated into our evaluation matrix, known as the SonoScore, specifically developed for this study. The SonoScore utilizes a Likert scale, allowing for the allocation of 1–5 points in various categories, including depth, gain, resolution, and detail. Each of these categories is critical in assessing the quality and accuracy of the ultrasound images produced by the students.

For the assessment of the 'correct setting,' a more binary scoring approach was employed. Students were allocated either one or three points based on the adequacy of the preset they selected. This binary scoring system aimed to simplify the evaluation of whether students had chosen the most appropriate settings for their ultrasound examinations.

**Fig. 3:**
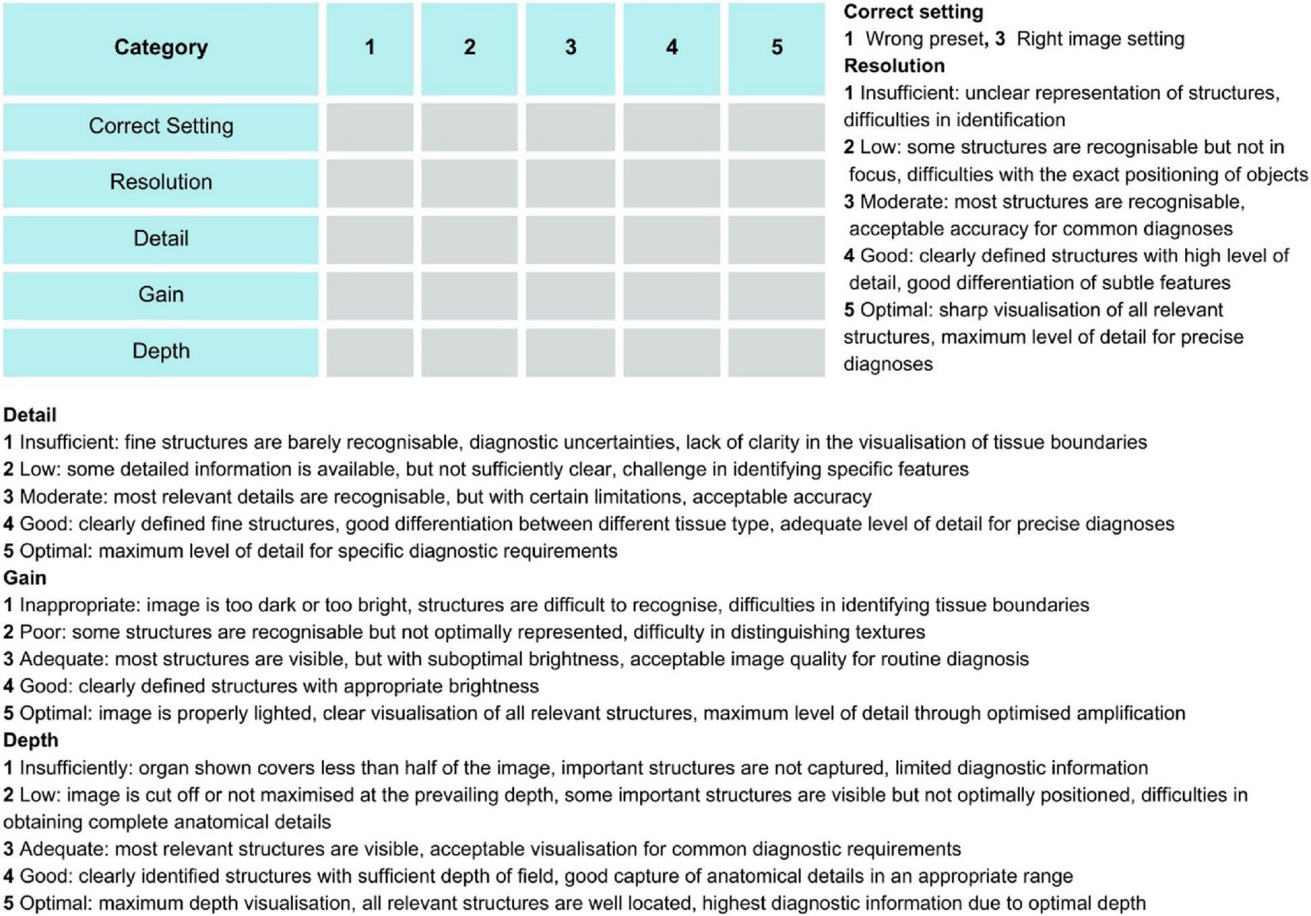
Image rating matrix and score breakdown of the SonoScore. The evaluation tool SonoScore developed for the course to assess the resulting ultrasound images is shown. A Likert scale was used and 1–5 points can be awarded for each of the categories such as depth, gain, resolution and detail and 1 or 3 points depending on the selection of the correct setting

A detailed breakdown of the respective scores in each category, as determined by the SonoScore, is provided in Fig. 3. This figure illustrates how points were distributed across the different evaluation criteria, offering insights into the areas where students excelled and those where further improvement was needed. The integration of the SonoScore into our assessment framework represents a significant step towards achieving more standardized and objective evaluations in ultrasound education.

The SonoScore system can be used across different organs for image evaluation in an educational setting. Accordingly, the SonoScore was used to evaluate the images uploaded to the Butterfly Cloud by the students from the courses and the resulting images of the lungs, bladder, spleen, kidneys, thyroid, aorta and vena cava were rated. At the end of the four months, the students were asked to participate in a voluntary survey specifically developed for the course to assess their use of the device (Supplementary file 1). By this point, the students had already begun their second tertial and most of them were no longer active in our clinic.

The local ethics committee of the University of Bonn approved the study (253/23-EP) and all enrolled students provided written informed consent to participate in the course and allow the use of their images. To manage incidental findings, we employed an article that offers a framework for identifying and addressing them within the context of ultrasound courses [16].

## Results

Forty students were provided with handheld ultrasound devices for a duration of four months. At the time, they were in the first of their three clinical rotations, having just commenced their practical year.

### Optional ultrasound courses participation and image rating

As participation in the course was voluntary and contingent upon the students' ward assignments and duties, the composition of the course varied weekly. Out of the 40 students provided with an ultrasound device, a minimum of 20 students attended each session. The first module, covering the introduction and the Focused Assessment with Sonography for Trauma (FAST), had the highest attendance with 29 participants, followed by the lung module with 27 participants.

Each organ was evaluated individually in the image rating, with specific focus on the categories of setting, resolution, detail, gain, and depth. The setting category had a maximum score of 3 points, while the other categories had a maximum score of 5 points. The ultrasound images of the lungs achieved the highest average score, with a mean of 18.82 points (SD ± 4.30) as shown in Table 1. In contrast, the images of the aorta and vena cava had the lowest average score, with a mean of 16.62 points (SD ± 1.55). The overall mean score for all evaluated images was 17.47 points (SD ± 2.74).

**Table 1:** Evaluation of the ultrasound course images generated during the voluntary course for students in their practical year. The average score achieved in the various categories of the image rating and the average total score are displayed, including the standard deviation. Means and standard deviations are presented

Organ	Setting	Resolution	Detail	Gain	Depth	Average total score
Lung	2.82 ± 0.57	4,00 ± 0.95	4.14 ± 1.10	3.64 ± 0.98	4.23 ± 1.13	18.82 ± 4.30
Bladder	3.00 ± 0.0	3.77 ± 0.95	3.59 ± 1.03	3.86 ± 0.92	3.36 ± 1.02	17.59 ± 2.79
Spleen	3.00 ± 0.0	3.50 ± 0.63	3.64 ± 0.81	3.86 ± 0.74	2.93 ± 0.80	16.93 ± 1.80
Kidney	3.00 ± 0.0	3.16 ± 0.63	3.48 ± 0.95	3.61 ± 0.66	3.32 ± 0.82	16.58 ± 1.93
Thyroid gland	2.22 ± 0.98	3.61 ± 0.57	4.26 ± 0.74	3.83 ± 0.82	4.17 ± 0.70	18.09 ± 1.93
Aorta and vena cava	3.00 ± 0.0	3.15 ± 0.53	3.38 ± 0.74	3.54 ± 0.75	3.54 ± 0.75	16.62 ± 1.55
Mean	2.82 ± 0.57	3.54 ± 0.81	3.77 ± 0.98	3.72 ± 0.83	3.62 ± 1.00	17.47 ± 2.74

### Survey responses

Out of the 40 students, 21 participated in the subsequent survey, representing a response rate of 52.5%. Among the respondents, 67% reported using the device independently four times or fewer over the course of the tertial (four months). Additionally, 9% of the students utilized the device biweekly, 14% used it weekly, and one student reported daily use of the device.

In terms of prior experience, 10 out of 21 students indicated that they had independently performed only 0–10 ultrasound examinations before starting their practical year. The majority of survey participants reported using the device for 0–1 h in their free time.

**Fig. 4:**
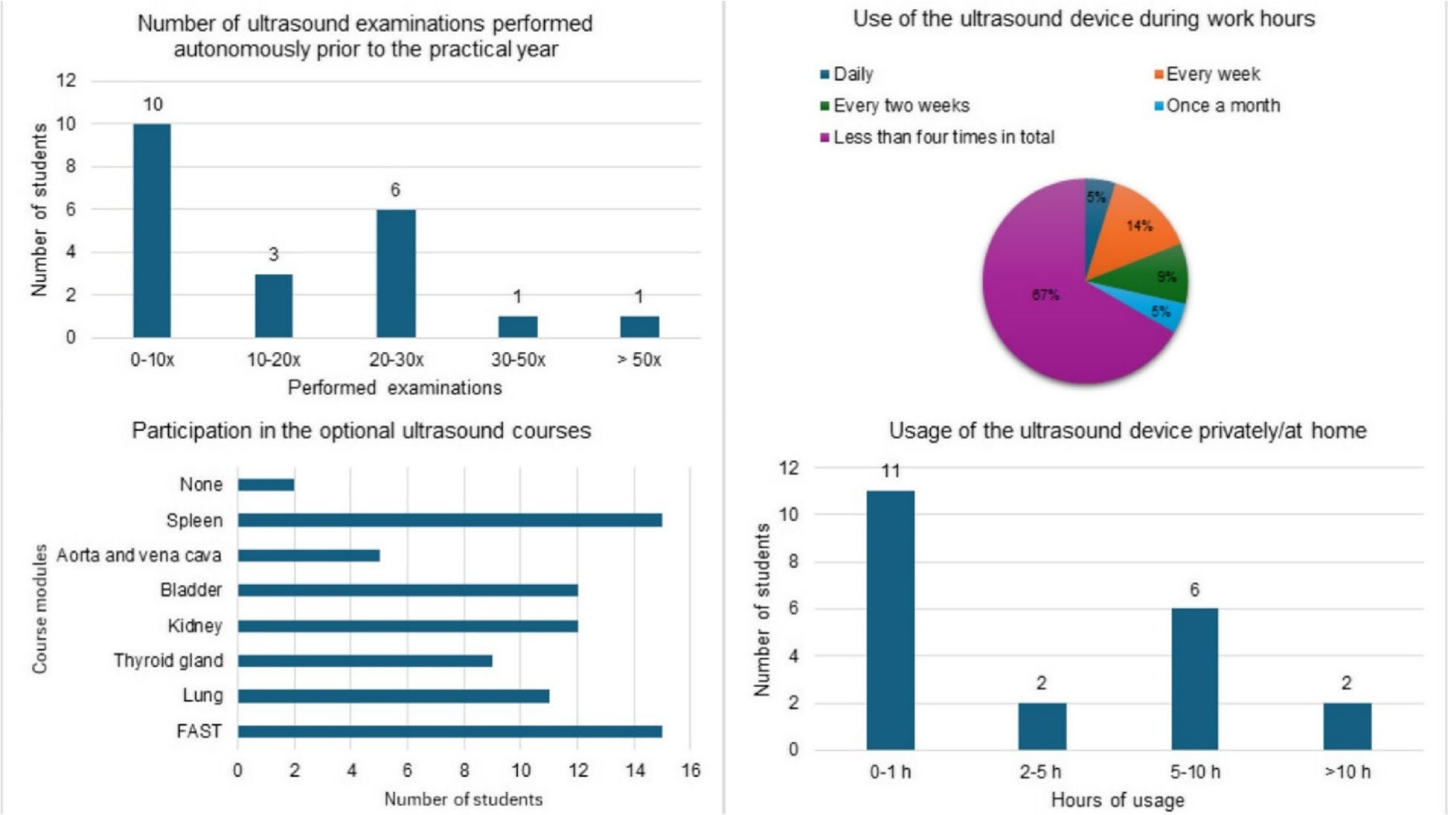
Participant data on previous ultrasound experience and use of the ultrasound devices. Four different graphics are shown, which are derived from the participant survey. At the top left, the number of ultrasound examinations performed independently prior to the practical year is displayed. Top right shows the use of the device during the practical year. The bottom left shows an overview of the voluntary courses attended. Bottom right illustrates the private usage of the device at home and during their freetime

The survey also included a section for comments and feedback, which students were explicitly encouraged to provide. One comment highlighted a student's difficulty in using the device within the anesthesia department. The student noted that the size of the iPad prevented its use in the operating theater, and personal devices were not permitted in the intensive care unit (Fig. 4).

Four students reported that they had problems with their login until the end of the tertial or did not receive a user ID at all resolving in the low usage. A separate person in each department was responsible for acquiring the user ID and solving issues.

## Discussion

The aim of this study was to further implement the BI-POCUS curriculum [13] with the objective of enhancing the ultrasound skills of students during their practical year. Other studies have distributed handheld devices to groups of first year students to enhance the teaching of anatomy [17] and different voices recommend the use and provision of handheld ultrasound devices for apprentices [18, 19].

The utilization of the ultrasound devices during working hours was markedly lower than anticipated, with the majority of students using the device only once a month or less frequently. The underlying reasons for this limited usage are not entirely clear and could be attributed to various factors, including potential technical difficulties or a lack of independence and confidence. Notably, most students had performed only 0–10 ultrasound examinations independently prior to the start of their practical year.

This feedback is particularly disheartening given the insufficient integration of ultrasound training into the medical curriculum and the growing advocacy, including from students, for enhanced ultrasound education. The limited use of the devices raises concerns about the justification of the effort, organization, and expenditure of material resources involved in providing ultrasound devices and iPads to students.

Comparable findings were observed in a related study involving internal medicine interns. In this study, interns were divided into two groups: one group received personal handheld ultrasound devices, while the other group did not. The results indicated that the interns who were provided with personal ultrasound devices did not show an improved ability to distinguish between normal and abnormal findings in image assessments. Additionally, there was no significant increase in the number of point-of-care ultrasound (POCUS) examinations conducted by this group.

These findings highlight the need for a reassessment of the current strategies for integrating ultrasound training into medical education. Despite the provision of advanced technological tools, the anticipated enhancement in practical skills and increased usage of ultrasound devices did not materialize. This suggests that simply providing access to technology is insufficient without comprehensive training, ongoing support, and effective integration into the clinical workflow. Future efforts should focus on addressing these gaps to ensure that students can fully utilize the tools provided and develop the necessary skills for proficient ultrasound use in clinical practice [20]. They concluded that, given the limited time trainees dedicate to direct patient care, providing portable ultrasound devices without sufficient guidance is inadequate for effective skill acquisition. A similar issue may have occurred in our program. Although optional ultrasound courses were offered, students lacked direct contact with mentors or supervisors within the various departments to guide or oversee their examinations on the ward.

Participation in the optional ultrasound courses was, however, satisfactory, with over 50% of students attending despite their ongoing ward duties. The average image rating of 17.47 ± 2.74 out of a possible 23 points indicates good performance in image acquisition. To thoroughly assess learning outcomes, pre- and post-course tests would be necessary to identify improvements in ultrasound techniques. Implementing such assessments would require substantial personnel resources.

To optimize the course structure, it should be enhanced to better introduce students to ultrasound imaging. Addressing the identified limitations, such as the lack of direct supervision and structured guidance, is crucial for future iterations of the course. Ensuring that students have access to dedicated mentors and more comprehensive training could significantly improve their skill acquisition and overall proficiency in ultrasound imaging.

### Limitations

This study has several limitations that need to be considered when interpreting the results. First, it was conducted at a single institution, which may limit the generalizability of the findings to other settings or medical schools. The sample size was relatively small, involving only forty students, which could affect the statistical power of the study and the robustness of the conclusions drawn. Another significant limitation is the inability to track the precise timing or location of the device usage. This lack of detailed usage data means that our understanding of how and when the devices were used is incomplete. Additionally, the usage rates reported by the interns are self-reported and may be subject to recall bias, potentially leading to inaccuracies in the data. The study also faced challenges with feedback collection. The survey conducted at the end of the tertial had a response rate of just over 50%, with 21 out of 40 students participating. This limited feedback may not fully represent the experiences and opinions of all students involved in the study. Furthermore, as the survey was carried out after the students had already started their next tertial, some may not have felt sufficiently motivated to participate, further skewing the response rate.

Technical issues also posed a problem during the study. Several students reported difficulties in obtaining their login credentials for the ultrasound devices, which hindered their ability to use the equipment effectively. Each department had a designated person responsible for managing login data, but there were inconsistencies in implementation, leading to significant discrepancies. This issue highlights the need for a more streamlined and centralized process for managing access to the devices.

The usage of the ultrasound devices during working hours was notably lower than expected. The majority of students reported using the device only once a month or even less frequently. This low utilization rate raises questions about the effectiveness of the program and the students' ability to incorporate ultrasound practice into their daily routines. Factors contributing to this low usage could include technical difficulties, a lack of prior experience (with most students having performed 0–10 ultrasound examinations before the practical year), or insufficient support and guidance in the clinical setting.

## Conclusions

The objective of this study was to advance the implementation of the BI-POCUS curriculum, with a focus on enhancing students' ultrasound skills during their practical year. However, the utilization of the ultrasound devices during working hours was markedly lower than anticipated, with the majority of students using the device only once a month or less frequently. The limited use of the devices raises concerns about the justification of the effort, organization, and expenditure of material resources. Future endeavours will involve technical enhancements, such as improving the provision of login data, as well as closer monitoring of device usage and learning progress to further advance the project.

Our findings contribute to the ongoing discourse on medical education reform, highlighting the importance of incorporating practical, hands-on training in ultrasound to prepare future healthcare professionals for their clinical roles. Ensuring that ultrasound becomes a fundamental component of medical training is essential, given its importance and utility in clinical practice.

### Supplementary Information

Supplementary Material 1

## Data Availability

Data are available upon request by the authors.

## Abbreviations

POCUS: Point-of-Care Ultrasound
BI-POCUS: Bonn Internship Curriculum for Point-of-Care Ultrasound
FAST: Focused Assessment with Sonography for Trauma

## Data availability

Data available on request from the authors.
