# Supplementary material for: First insights of integrating the Bonn Internship Curriculum for Point-of-Care Ultrasound (BI-POCUS): progress and educational aspects
Source: BMC Med Educ. 2024 Aug 19;24:894. doi: 10.1186/s12909-024-05904-2 (PMC11334524; doi:10.1186/s12909-024-05904-2)
Supplement: Supplementary file 1 — Supplementary Material 1 [file 12909_2024_5904_MOESM1_ESM.docx]

How often did you carry out ultrasound examinations independently before your final year?

1. 0-10 times
2. 10-20 times
3. 20-30 times
4. 30-50 times
5. >50 times

How often did you use the ultrasound machine during your tertial?

1. Daily
2. 2-3 times a week
3. Once a week
4. Every two weeks
5. Once a month
6. Less than 2-3 times in total

Did you use the ultrasound device privately/at home?

1. 0-1 hour
2. 2-5 h
3. 5-10 h
4. >10 h

Which optional ultrasound courses did you take part in?

1. Introduction/FAST
2. Lungs
3. Thyroid gland
4. Kidney and urinary bladder/spleen
5. Aorta and vena cava
6. None

What examinations did you use the device for during working hours?

1. FAST
2. Kidney and urinary bladder
3. Spleen
4. Aorta/cava/fluid status
5. Pancreas
6. Lung/pleura
7. Thyroid gland
8. Joints/tissues
9. Vessels/exclusion of thrombosis
10. Other (please name)

Which of these examinations did you carry out under supervision?

1. FAST
2. Kidney/urinary bladder
3. Spleen
4. Aorta/cava/fluid status
5. Pancreas
6. Lung/Pleura
7. Thyroid gland
8. Joints/tissues
9. Vessels/thrombosis
10. Other
